# Efficacy of tocilizumab in patients with COVID-19 ARDS undergoing noninvasive ventilation

**DOI:** 10.1186/s13054-020-03306-6

**Published:** 2020-09-29

**Authors:** Francesco Menzella, Matteo Fontana, Carlo Salvarani, Marco Massari, Patrizia Ruggiero, Chiara Scelfo, Chiara Barbieri, Claudia Castagnetti, Chiara Catellani, Giorgia Gibellini, Francesco Falco, Giulia Ghidoni, Francesco Livrieri, Gloria Montanari, Eleonora Casalini, Roberto Piro, Pamela Mancuso, Luca Ghidorsi, Nicola Facciolongo

**Affiliations:** 1 Pneumology Unit, Arcispedale Santa Maria Nuova, Azienda USL-IRCCS di Reggio Emilia, 42123 Reggio Emilia, Italy; 2 Division of Rheumatology, Arcispedale Santa Maria Nuova, Azienda USL-IRCCS di Reggio Emilia, Reggio Emilia, Italy; 3 University of Modena and Reggio Emilia, Modena, Italy; 4 Infectious Disease Unit, Arcispedale Santa Maria Nuova, Azienda USL-IRCCS di Reggio Emilia, Reggio Emilia, Italy; 5 Epidemiology Unit, Azienda USL-IRCCS di Reggio Emilia, Reggio Emilia, Italy

**Keywords:** SARS-CoV-2, COVID-19, Acute respiratory distress syndrome, Tocilizumab, Noninvasive ventilation

## Abstract

**Background:**

The severity of severe acute respiratory syndrome coronavirus 2 (SARS-CoV-2) infection is extremely variable, ranging from asymptomatic patients to those who develop severe acute respiratory distress syndrome (ARDS). As for now, there are still no really effective therapies for coronavirus disease 2019 (COVID-19). Some evidences suggest that tocilizumab (TCZ) may avoid the progression of severe COVID-19. The aim of this retrospective case-control study was to analyze the efficacy and safety of TCZ in patients with COVID-19 ARDS undergoing noninvasive mechanical ventilation (NIV).

**Methods:**

Seventy-nine consecutive patients with severe COVID-19 pneumonia and worsening acute respiratory failure (ARF) were admitted to the Pulmonology Unit of Azienda USL of Reggio Emilia-IRCCS. All patients were inflamed (elevated CRP and IL-6 levels) and received NIV at admission according to the presence of a pO_2_/FiO_2_ ratio ≤ 200 mmHg. The possibility of being treated with TCZ depended on the drug availability. The primary outcome was the in-hospital mortality rate. A secondary composite outcome of worsening was represented by the patients who died in the pulmonology unit or were intubated.

**Results:**

Out of 79 patients, 41 were treated with TCZ. Twenty-eight patients received intravenous (IV) TCZ and 13 patients received subcutaneous (SC) TCZ. In-hospital overall mortality rate was 38% (30/79 patients). The probabilities of dying and being intubated during the follow-up using Kaplan-Meier method were significantly lower in total patients treated with TCZ compared to those of patients not treated with TCZ (log-rank *p* value = 0.006 and 0.036, respectively). However, using Cox multivariate analyses adjusted for age and Charlson comorbidity index only the association with the reduced risk of being intubated or dying maintained the significance (HR 0.44, 95%CI 0.22–0.89, *p* = 0.022). Two patients treated with TCZ developed cavitating lung lesions during the follow-up.

**Conclusions:**

This study shows that TCZ treatment may be effective in COVID-19 patients with severe respiratory impairment receiving NIV. More data on safety are required. Randomized controlled trials are needed to confirm these results.

## Background

The severity of severe acute respiratory syndrome coronavirus 2 (SARS-CoV-2) infection is extremely variable, ranging from asymptomatic patients to those who develop pneumonia with acute respiratory distress syndrome (ARDS) [1]. The unpredictability of clinical evolution probably depends on viral strain and load, age, and comorbidities [2]. Individual susceptibility of the host may probably modulate the severity of response, in particular cytokine storm, that in some cases evolves to multiple organ dysfunction syndrome and death [3].

In SARS-CoV infections, elevated levels of serum pro-inflammatory cytokines (IFN-γ, IL-1, IL-6, IL-12, and TGFβ) and chemokines (CCL2, CXCL10, CXCL9, and IL-8) have been detected in patients with severe forms compared to less complicated cases [4]. Even in Middle East respiratory syndrome (MERS) infection, the presence of elevated levels of serum pro-inflammatory cytokines (IL-6 and IFN-α) and chemokines (IL-8, CXCL-10 and CCL5) is documented in the most severe forms compared to the milder ones [5]. With regard to SARS-CoV-2, the most commonly elevated cytokines were IL2, IL-6, IL7, IL10, GSCF, IP10, MCP1, MIP1a, and TNF α, the levels of which were higher in the more compromised patients [2, 6]. It is important to note that the levels of IL-6 in the blood of the patients with severe disease were higher than those observed in patients with milder disease and an association between elevated IL-6 serum levels and increased mortality rates was found [6, 7].

Except glucocorticoids (GCs), there are still no really effective therapies for coronavirus disease 2019 (COVID-19) [8]. Some evidences suggest that tocilizumab (TCZ), a humanized anti-interleukin 6 (IL-6) receptor monoclonal antibody (mAb), may be effective and avoid the progression of severe COVID-19 [9–13].

The aim of this retrospective case-control study was to analyze the efficacy and safety of TCZ in a cohort of patients with COVID-19 ARDS undergoing noninvasive mechanical ventilation (NIV).

## Patients and methods

Seventy-nine consecutive patients with severe COVID-19 pneumonia and worsening acute respiratory failure (ARF) were admitted to the Pulmonology Unit of Azienda USL-IRCCS of Reggio Emilia between March 10, 2020, and April 14, 2020, and were followed-up until May 18, 2020 (median follow-up, 60 days; interquartile range, 22–69 days). All patients (71% males) had a SARS-CoV-2 infection confirmed by a positive reverse-transcriptase polymerase chain-reaction (RT-PCR) assay for SARS-CoV-2 in a respiratory tract specimen and clinical and radiological findings compatible with COVID-19 severe pneumonia (demographic and clinical characteristics are shown in Table 1). Basic laboratory profile included IL-6, C-reactive protein (CRP), ferritin, D-dimer, lactate dehydrogenase (LDH) levels, and complete blood count (CBC) (Table 2). Serum CRP and IL-6 levels at admission were elevated in all patients with mean + SD values of 11.9 ± 7.2 mg/dl and 147.2 ± 180.4 pg/ml, respectively, indicating the hyperinflammatory state of most patients. All 79 patients needed NIV at admission according to severity of ARF based on the ratio of arterial partial pressure of oxygen to fraction of inspired oxygen (PaO_2_/FiO_2_) cut-off values recorded at ARDS onset. NIV was used preferentially for moderate-severe ARF due to availability and exercise in NIV in our unit.

**Table 1:** Characteristics of the 79 patients and comparisons between patients treated with tocilizumab + Std therapy and those treated with Std therapy alone

	Overall population	Tocilizumab + Std therapy	Std therapy	***p*** value
**Demographics and clinical features**				
No. of patients	79	41	38	
Age (years), mean ± SD	66.5 ± 11.4	63.3 ± 10.6	70.3 ± 11.3	0.005
Male, *n* (%)	56 (71)	29 (71)	27 (71)	0.9
Smokers, *n* (%)	3 (4)	2 (5)	1 (3)	0.9
Former smokers, *n* (%)	22 (28)	12 (29)	10 (26)	0.8
Non-smokers, *n* (%)	54 (68)	27 (66)	27 (71)	0.6
BMI, mean ± SD	29.7 ± 5.2	30.6 ± 5.3	28.4 ± 4.7	0.1
Number of comorbidities, mean ± SD	2.9 ± 2.1	2.6 ± 1.7	3.2 ± 2.4	0.2
Charlson Comorbidity index, mean ± SD	3.4 ± 2.2	2.7 ± 2.1	4.2 ± 2.2	0.002
SOFA index at admission, mean ± SD	4.3 ± 1.3	4 ± 1.1	4.6 ± 1.5	0.07
Not eligible for ICU, *n* (%)	18 (23)	5 (12)	13 (34)	0.03
Respiratory rate (breaths/min), mean ± SD				
At admission	24.6 ± 4.9	24.8 ± 4.7	24.4 ± 5.3	0.9
At 72 h	25.6 ± 7.3	25.8 ± 6.8	25.5 ± 8.1	0.8
At 7 days	21.3 ± 5.8	19.8 ± 2.6	22.4 ± 7.3	0.5
**Outcomes**				
Intubation/death, *n* (%)	41 (52)	16 (39)	25 (64)	0.02
Death, *n* (%)	30 (38)	10 (24)	20 (53)	0.01
**Treatment**				
Hydroxychloroquine, *n* (%)	75 (95)	41 (100)	34 (89)	0.05
Antivirals (lopinavir/ritonavir or darunavir/cobicistat), *n* (%)	41 (52)	20 (49)	21 (55)	0.7
Anticoagulants (full dosage), *n* (%)	20 (25)	10 (24)	10 (26)	0.9
Steroids (methylprednisolone 0.5–1 mg/kg/die)	55 (70)	28 (68)	27 (71)	0.9
Tocilizumab, *n* (%)	41 (52)			
IV, *n* (%)	28 (36)			
SC, *n* (%)	13 (16)

**Table 2:** Blood tests of the 79 patients and comparisons between patients treated with tocilizumab + Std therapy and those treated with Std therapy alone

	Overall population	Tocilizumab + Std therapy	Std therapy	***p*** value
**pH, mean ± SD**				
At admission	7.45 ± 0.05	7.46 ± 0.5	7.45 ± 0.05	0.66
At 72 h	7.44 ± 0.07	7.44 ± 0.03	7.43 ± 0.1	0.07
At 7 days	7.45 ± 0.04	7.44 ± 0.04	7.46 ± 0.03	0.05
**pO**_**2**_ **(mmHg), mean ± SD**				
At admission	67.4 ± 20.3	67.1 ± 16.9	67.7 ± 23.8	0.66
At 72 h	85.7 ± 29.9	85.2 ± 18.5	86.1 ± 39.8	0.4
At 7 days	100.8 ± 42.7	104 ± 42.9	95.9 ± 43.1	0.5
**pCO**_**2**_ **(mmHg), mean ± SD**				
At admission	36.5 ± 6.2	36.9 ± 5.5	36 ± 7	0.3
At 72 h	40.7 ± 11.1	40.2 ± 4.8	41.4 ± 15.6	0.4
At 7 days	40 ± 5.9	39.4 ± 4.9	40.8 ± 7.2	0.5
**pO**_**2**_**/FiO**_**2**_ **ratio (mmHg), mean ± SD**				
At admission	120.1 ± 41.6	117.8 ± 34.4	122.8 ± 48.9	0.8
At 72 h	155.6 ± 78.6	143.8 ± 53	169.6 ± 100.2	0.6
At 7 days	191 ± 86.8	186.2 ± 78.5	198.1 ± 99.3	0.8
**Leukocytes (10**^**9**^**/L), mean ± SD**				
At admission	6.42 ± 2.68	6.2 ± 2.7	7.2 ± 2.4	0.1
At 72 h	8.1 ± 3	8.2 ± 3.2	7.6 ± 2.2	0.9
At 7 days	8.3 ± 3.9	8.2 ± 4.1	8.5 ± 3.3	0.8
**Neutrophils (10**^**9 /**^ **L), mean ± SD**				
At admission	4.77 ± 1.89	4.6 ± 1.9	5.8 ± 1.7	0.07
At 72 h	6.8 ± 3	6.9 ± 3.1	6.4 ± 2.6	0.6
At 7 days	6.5 ± 4.2	6.4 ± 4.4	6.7 ± 3.6	0.7
**Lymphocytes (10**^**9 /**^ **L), mean ± SD**				
At admission	0.8 ± 0.35	0.8 ± 0.4	0.9 ± 0.3	0.6
At 72 h	0.8 ± 0.5	0.8 ± 0.5	0.8 ± 0.5	0.9
At 7 days	1.2 ± 0.5	1.2 ± 0.6	1.2 ± 0.4	0.8
**D-dimer (ng/dl), mean ± SD**				
At admission	774.5 ± 917	775 ± 957.8	–	NA
At 72 h	6388.2 ± 8143.4	5388.3 ± 8143.4	–	NA
At 7 days	2602.7 ± 2861.1	2602.7 ± 2861.1	–	NA
**Ferritin (ng/ml), mean ± SD**				
At admission	688.9 ± 683.6	652.9 ± 721.6	–	NA
At 72 h	1758.9 ± 1545.7	1906 ± 1603	830 ± 415	0.3
At 7 days	1137.9 ± 881.6	1194 ± 903.4	577 ± 349.3	0.4
**CRP (mg/dl), mean ± SD**				
At admission	11.9 ± 7.2	11.2 ± 7	14.3 ± 7.6	0.2
At 72 h	8.5 ± 9.5	6.4 ± 6.5	17.2 ± 14.9	0.02
At 7 days	4.5 ± 8.4	2.3 ± 4.9	13.3 ± 13.5	0.001
**LDH (U/l), mean ± SD**				
At admission	687.7 ± 244.6	700.5 ± 250.5	534 ± 24.4	0.2
At 72 h	745.3 ± 320.3	757 ± 314	701 ± 361.7	0.2
At 7 days	682.3 ± 326.5	693.1 ± 334.9	633.3 ± 309.1	0.7
**IL-6 (pg/ml), mean ± SD**				
At admission	147.2 ± 180.4	145.9 ± 190.1	–	NA
At 72 h	656.4 ± 878.9	656.4 ± 878.9	–	NA
At 7 days	623.2 ± 1217.4	623.2 ± 1217.4	–	NA

*Std therapy* standard therapy, *CRP* C-reactive protein, *LDH* lactate dehydrogenase, *IL-6* interleukin-6, *NA* not applicable

All patients had a PaO_2_/FiO_2_ ratio > 100 and ≤ 200 mmHg despite oxygen delivered through a Venturi mask (FiO_2_ 60%). Table 2 shows that the mean ± SD PaO_2_/FiO_2_ ratio in our patients at admission was 120.1 ± 41.6 mmHg. NIV was provided using Philips V680™ (Respironics INC®, Pennsylvania, USA) or Hamilton G-5 (Hamilton Medical AG, Bonaduz, Switzerland) mechanical ventilators through a full-face mask. Table 1 also shows the other characteristics of the patients at admission.

Of 79 patients included in the study, 41 were treated with TCZ. The decision to treat the patients with TCZ and the formulation of the drug we used were strictly based on drug availability. The patients were enrolled from March 11 to April 14, 2020, and were followed until May 25 (the last enrolled patients had a follow-up of 31 days). Subcutaneous (SC) TCZ was administered because intravenous (IV) TCZ was not available for a period of time; furthermore, we also did not have available both formulations at the end of March and at the beginning of April (March 20–April 5, 2020) for a period of 16 days. Therefore, all 38 patients who started NIV in this period were not treated with TCZ and they represented the control group. Patients were treated with TCZ at the beginning of NIV. The primary outcome was the in-hospital mortality rate. A secondary composite outcome of no response to TCZ was represented by the patients who were intubated or died during the hospitalization in the pulmonology unit. To obtain data on infections, one of the authors (MF) revised the medical records of all patients. Only infections occurring after administration of TCZ were included in the treatment group.

Improvement was considered when the following conditions were satisfied: increase in the PaO_2_/FiO_2_ ratio of 30% after NIV, FiO_2_ < 50%, respiratory rate (RR) < 30 breaths per minute, expiratory tidal volume >5 ml/kg body weight expected with a pressure support < 10 cm H_2_O and PEEP < 8 cm H_2_O. In this case, NIV was progressively suspended and a Venturi oxygen mask with variable FiO_2_ was started on the basis of arterial blood gas data (Table 2).

NIV failure was defined according to ERS/ATS guidelines [14]. The decision to intubate a patient was made on the basis of the following criteria: persistent or worsening of acute respiratory failure (ARF) (PaO_2_/FiO_2_ ratio < 100 mmHg, respiratory rate > 36/min) despite NIV, development of conditions requiring endotracheal intubation (EOT) in order to protect airways (coma or convulsive disorder) or to manage abundant tracheal and/or bronchial secretions, or hemodynamic or electrocardiographic instability.

This study was approved by the Local Ethics Committee of Area Vasta Emilia Nord (no. 855/2020/OSS/AUSLRE) and conducted according to the principles of the Declaration of Helsinki. Since not all patients were able to give their informed consent, the Ethics Committee waived this requirement. Informed consent was sought from all surviving patients as soon as they regained their mental competence. This study has been partially funded by the Italian Ministry of Health, grant number COVID-2020-12371808. The funder had no role in the definition of the study design neither in the interpretation and publication of the results.

### Statistical analysis

Descriptive statistics for continuous variables were presented as mean ± standard deviation (SD). The Welch’s *t* test and the Mann-Whitney test were used for comparison of two groups, while the Brown-Forsythe and Welch ANOVA test and nonparametric Kruskal-Wallis test were used for comparison of three or more continuous variables, where appropriate. Chi-square test and Fisher exact test were used for comparison of categorical variables. The impact of TCZ on mortality and on the probability of dying or being intubated was assessed by Kaplan-Meier survival function estimates, and the differences between treatment and standard therapy were analyzed with the log-rank test. The Cox proportional hazards model was used to assess the relation between tocilizumab treatment, age, and Charlson comorbidity index at diagnosis and mortality and intubation/death. We reported age and Charlson comorbidity index-adjusted hazard ratios (HRs) and 95% confidence intervals. A *p* value less than 0.05 was considered significant. Statistical analysis was performed using SPSS version 22.0 (IBM Statistics, Armonk, NY: IBM Corp, USA).

## Results

Out of 79 patients, 41 were treated with TCZ. Twenty-eight patients received IV TCZ (8 mg/kg—max 800 mg) by two consecutive infusions 12 h apart. Thirteen patients received SC TCZ ranging from 2 to 4 doses of 162 mg TCZ (administered simultaneously in a different injection site) per patient depending on drug availability and body weight, according to an internal protocol developed due to a temporary unavailability of IV formulation. Pharmacokinetically, 2 doses of 162 mg of SC TCZ administered simultaneously are equivalent to 4 mg/Kg IV [15, 16].

Table 1 shows the characteristics of the patients and concomitant treatments during the follow-up. In the TCZ treatment group, CRP levels were significantly lower at 72 h and 7 days after starting therapy compared with the standard therapy group (*p* = 0.02 and *p* = 0.001, respectively), while an increase in IL-6 levels was found at the same times, due to the known effect of TCZ that blocking IL-6 receptors increases the serum levels of free IL-6. At admission, patients treated with TCZ were younger, had a lower Charlson comorbidity index, and received more frequently hydroxychloroquine compared to the standard therapy group (*p* = 0.005, *p* = 0.002, and *p* = 0.05, respectively); however, the other evaluated parameters were not different, including the number of comorbidities and SOFA index.

CRP levels determined before TCZ administration (T0) and after 3 (T3) and 7 (T7) days were not significantly different between patients treated with IV TCZ and SC TCZ (11.2 ± 7.1 mg/dl vs 11.2 ± 7.5, *p* = 0.77; 5.9 ± 5.9 mg/dl vs 7.6 ± 7.9, *p* = 0.80; 1.1 ± 1.5 mg/dl vs 5.1 ± 8.5, *p* = 0.78; respectively).

**Fig. 1:**
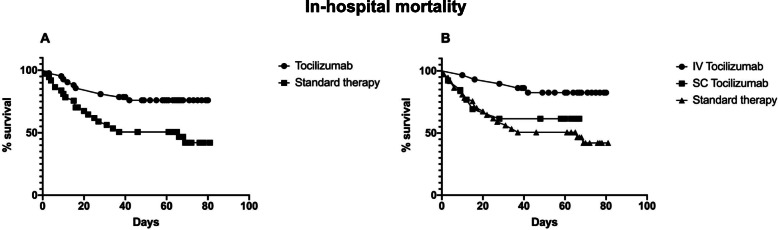
**a** Differences in survival during the follow-up in noninvasively ventilated patients treated and not treated with TCZ (log-rank *p* value = 0.0057). **b** Differences in survival during the follow-up between patients treated with IV or SC TCZ, or not treated with TCZ. No significant differences were found between SC and IV TCZ -treated patients (log-rank *p* value = 0.092), and the differences between IV TCZ-treated and untreated patients was significant (log-rank *p* value = 0.0017), but not the difference between SC TCZ-treated and untreated patients (log-rank *p* value = 0.53)

In-hospital mortality rate was 38% (30/79 patients). Regarding all TCZ-treated patients, mortality rate was 24% (10/41 patients), while was 18% (5/28 patients) in the IV TCZ subgroup, 38% (5/13 patients) in the SC TCZ subgroup, and 53% (20/38 patients) in the subgroup of patients not treated with TCZ. The differences between the 3 subgroups were statistically significant (*p* = 0.0085). Figure 1 shows Kaplan-Meier estimates for the probability of dying during the follow-up. The probability of dying of all TCZ-treated patients was significantly lower compared to that of patients not treated with TCZ (log-rank *p* value = 0.0057). No significant differences were found between SC and IV TCZ-treated patients (log-rank *p* value = 0.092), the differences between IV TCZ-treated and untreated patients was significant (log-rank *p* value = 0.0017), but not the differences between SC TCZ-treated and untreated patients (log-rank *p* value = 0.53). However, last comparisons were limited by the low number of patients evaluated.

**Fig. 2:**
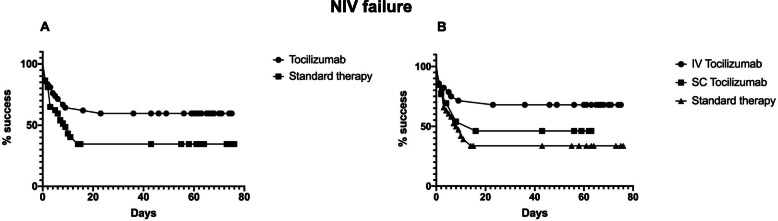
**a** Differences in probability of dying in the pulmonology unit or being intubated during the follow-up in noninvasively ventilated patients treated and not treated with TCZ (log-rank *p* value = 0.036). **b** Differences in probability of dying in the pulmonology unit or being intubated between patients treated with IV or SC TCZ, or not treated with TCZ. The difference was significantly lower in patients treated with IV TCZ compared to those untreated (log-rank *p* value = 0.01), but not in patients treated with the SC formulation (log-rank *p* value = 0.45)

Sixteen/41 (39%) patients treated with TCZ were intubated or died in the pulmonology unit: 9/28 (32%) treated with IV TCZ, 7/13 (54%) treated with SC TCZ, and 25/38 (66%) not treated with TCZ, respectively. The differences between the 3 subgroups were statistically significant (*p* = 0.037). The intubation rate was 7/28 in the IV TCZ group (25%), 2/13 in the SC TCZ group (15%), and 12/38 in the standard care group (31.6%, *p* = 0.507). Figure 2 shows Kaplan-Meier estimates for the probability of dying or being intubated during the follow-up. The probability of dying or being intubated was significantly lower in total patients treated with TCZ compared to those untreated (log-rank *p* value = 0.036). The difference was significantly lower in patients treated with IV TCZ (log-rank *p* value = 0.01), but not in patients treated with the SC formulation (log-rank *p* value = 0.45) compared to the untreated patients. At multivariate Cox proportional hazards analyses adjusted by sex and age, patients treated with tocilizumab had a significantly reduced risk of intubation or death during the follow-up period (HR 0.44, 95%CI 0.22–0.89, *p* = 0.022), while they did not have a reduced mortality (HR 0.55, 95%CI 0.22–1.35, *p* = 0.192).

A baseline CRP level of 10 mg/dl was selected to evaluate TCZ-treated patients according to their inflammatory status. Twenty-one patients had baseline CRP levels > 10 mg/dl (mean + SD, 17.0 + 5.0 mg/dl) and 20 patients < 10 mg/dl (5.2 + 2.4 mg/dl). No differences, neither for mortality nor for intubation/death, were observed between patients with baseline CRP > 10 mg/dl and those with values < 10 mg/dl (5/21, 24% vs 5/20, 25%, *p* = 0.90; and 7/21, 33.3% vs 9/20, 45.0%, *p* = 0.75, respectively).

Among the patients treated with TCZ, two developed cavitating lung lesions: *Staphylococcus aureus* was isolated in the first patient, while no infectious agent was identified at cultural examination in the second. However, antibiotic treatment resolved the lesions in both patients.

## Discussion

In this study, we evaluated the effectiveness of TCZ in a cohort of COVID-19 critically ill patients treated with NIV. Notably, pre-ventilation PaO_2_/FiO_2_ ratio was similar to a cohort of invasively ventilated patients in intensive care unit (ICU) [17], therefore with a high level of impairment.

The mortality rate and the probability of being intubated or dying in the pulmonology unit using Kaplan-Meier method were significantly lower in patients treated with TCZ compared to those who were not treated. However, when we used Cox multivariate analyses, adjusting for age and Charlson comorbidity index, only the association with the reduced risk of being intubated or dying in our ward maintained the significance, because the association between TCZ treatment and reduced mortality risk was no longer significant. These results suggest that TCZ could be an effective therapeutic option for the treatment of critically ill COVID-19 patients receiving NIV; however, more data are needed to confirm the efficacy of TCZ in this group of patients, particularly from randomized controlled trials.

We also evaluated whether patients with a more inflammatory phenotype could have a different response to TCZ. We selected a baseline CRP level of 10 mg/dl as the best cut-off because in several studies CRP values higher than 10 mg/dL identified the patients with more severe inflammation [18, 19]. However, we did not see any difference in TCZ-treated patients, neither for mortality nor for intubation/death between patients with baseline CRP ≥ 10 mg/dl and those with values < 10 mg/dl.

Due to a shortage of IV formulation, we administered SC TCZ. However, this formulation did not completely mimic the pharmacodynamic activity and the dosage of the IV formulation, even if we used SC dosages higher compared to those used in rheumatoid arthritis and other inflammatory conditions. It is important to note that the SC route takes some days to reach the peak plasma concentration after a single dose. This is due to the slow absorption through the lymphatic system into the systemic circulation [15, 16]. To overcome these limitations, the patients were simultaneously treated with multiple separate injections of SC TCZ. This strategy was supported from the findings of a comparative pharmacokinetic/pharmacodynamic study of SC vs IV TCZ [15]. We did not observe significant differences in the mortality rate and in the proportion of patients who were intubated or died in the pulmonology unit between patients treated with SC TCZ and those untreated; however, possible differences in efficacy between the two formulations of TCZ in COVID-19 ARDS must be confirmed by larger studies. The small number of the patients treated with SC formulation may have influenced the negative results. In the literature, there are reports demonstrating the efficacy of SC TCZ in reducing the mortality of severe COVID-19 pneumonia [13]. We also observed at T3 and T7 a similar reduction of CRP levels in patients treated with IV and SC TCZ, confirming at the administered doses a similar anti-inflammatory activity of the two TCZ formulations.

After the initial viral phase, some patients with COVID-19 develop a hyperinflammatory phase, which is manifested with biological changes of cytokine storm, usually associated with a quick respiratory deterioration due to pneumonitis. In these patients, glucocorticoids at high doses and/or IVs TCZ have become the treatment of choice in real life [20]. Although some retrospective and observational studies using TCZ in severe COVID-19 have shown promising results [9–13], other studies, including the preliminary results of randomized trials, and a systemic review and meta-analysis did not demonstrate a benefit for TCZ in improving clinical status or reducing the risk of ICU admission and mortality [21, 22]. However, TCZ may be effective in particular subgroups of patients, as we showed in this study in patients underlying NIV or Somers et al. in patients requiring invasive mechanical ventilation [23]. In a recent randomized trial, the use of dexamethasone in patients hospitalized with COVID-19 resulted in lower 28-day mortality among patients who were receiving either invasive mechanical ventilation or oxygen alone at randomization, but not among those receiving no respiratory support [8]. Therefore, the combined use of TCZ and GCs may be useful in patients with severe COVID-19 pneumonia to prevent invasive mechanical ventilation and/or death. In this regard, a recent prospective study demonstrated that a therapeutic strategy constituted by a course of high dose methylprednisolone, followed by tocilizumab if needed, may accelerate respiratory recovery, lower hospital mortality, and reduce the likelihood of invasive mechanical ventilation in COVID-19-associated cytokine storm syndrome [24]. A treatment with high-dose glucocorticoids as first therapeutic choice may also be convenient since glucocorticoids are safe, widely available and inexpensive.

The risk of severe infection in patients with COVID-19 undergoing noninvasive ventilation and treated with TCZ is not well defined. Somers et al. demonstrated that superinfections were common in patients with severe COVID-19 treated with TCZ and requiring invasive mechanical ventilation [23]. In this study, patients who received TCZ were more than twice as likely to develop a superinfection than untreated controls, driven primarily by a large increase in ventilator-associated pneumonia. *Staphylococcus aureus* accounted for ~ 50% of bacterial pneumonia. We observed cavitating lung lesions in 2/41 (4.8%) patients treated with TCZ, while none of such lesions were observed in the untreated patients. *Staphylococcus aureus* was isolated in the first patient, while no infectious agent was identified at cultural examination in the second; however, antibiotic treatment resolved the lesions in both patients. Probably, our study underestimates the incidence of severe infections; however, all secondary infections were accurately reviewed to ensure careful reporting. Furthermore, differently from Somers et al. study, we have enrolled noninvasively ventilated patients who are less exposed to superinfections compared to those receiving invasive mechanical ventilation.

Main limitations of this study are the retrospective nature and the relatively small number of patients enrolled. An important observation to report is that humidified high-flow nasal cannula (HHFNC) was not routinely used in our hospital because of shortage of devices during the first period of the pandemic. Furthermore, we preferentially use NIV for our better experience on this technique. For these reasons, NIV has been utilized in patients with a moderate to severe disease in our Unit as the first choice. On the other hand, during the outbreak, there was also a low ICU bed availability, having some difficulties in treating many patients with mechanical ventilation. Another limitation is that TCZ route of administration was heterogeneous and there are not definitive data regarding similarities in effectiveness between IV and SC formulations, considering the different pharmacokinetic characteristics. Also differences in ICU strain with restrictions on ICU access during the study period, differences in the severity of COVID-19 pneumonia at admission, in comorbidities and age could have influenced patient mortality and then the positive results of TCZ. However, multivariate analysis was corrected by age and comorbidity index and the only factor influencing the treatment choice was drug availability. Strength of the study was that all patients were homogeneously followed-up using a common standardized protocol. Furthermore, in Reggio Emilia-hospitalized COVID-19 patients, the mortality rate reached a plateau 1 month after hospitalization [25]; therefore, our study had an adequate follow-up period (median was 60 days) to properly observe COVID-19 related deaths.

## Conclusions

In summary, our preliminary data show that TCZ is effective in COVID-19 patients with severe respiratory impairment underlying NIV with a significant reduction in the proportion of patients who died in the pulmonology unit or were intubated. This may represent a subgroup of patients in which TCZ is particularly effective. However, regarding safety, in particular the increased risk of developing superinfections, more data are needed. Multicenter randomized controlled studies enrolling large series of patients are needed to confirm the efficacy and safety of TCZ in COVID-19 patients with respiratory involvement of different severity levels and to evaluate if there are differences in efficacy between SC and IV formulation of this drug.

## Data Availability

Data are however available from the authors upon reasonable request.

## Abbreviations

SARS-CoV-2: Severe acute respiratory syndrome coronavirus 2
ARDS: Acute respiratory distress syndrome
MERS: Middle East respiratory syndrome
TCZ: Tocilizumab
IL-6: Interleukin 6
mAb: Monoclonal antibody
NIV: Noninvasive mechanical ventilation
RT-PCR: Polymerase chain-reaction
CRP: C-reactive protein
LDH: Lactate dehydrogenase
CBC: Complete blood count
ARF: Acute respiratory failure
PaO_2_/FiO_2_: Arterial partial pressure of oxygen to fraction of inspired oxygen
SC: Subcutaneous
IV: Intravenous
RR: Respiratory rate
EOT: Endotracheal intubation
HHFNC: Humidified high-flow nasal cannulae
